# Short-term outcomes of anterior approach sacrospinous ligament fixation for apical vaginal prolapse - A retrospective study

**DOI:** 10.52054/FVVO.13.2.015

**Published:** 2021-06-28

**Authors:** S Siddiqui, A Gayen, V Wong

**Affiliations:** Brighton and Sussex University Hospital NHS Trust.

**Keywords:** Sacrospinous fixation, sacrospinous hysteropexy, apical prolapse, anterior approach SSF

## Abstract

**Introduction:**

Vaginal sacrospinous fixation and sacrospinous hysteropexy (SSF/SSHP) are highly effective procedures for apical compartment prolapse. The established technique is the posterior vaginal approach. The alternative anterior approach through an anterior vaginal incision, although occasionally mentioned in the literature, is less well established. However, this approach is a more appropriate route if posterior vaginal surgery is not indicated. The aim of this paper is to review surgical outcomes of anterior approach in our centre and to compare outcomes of SSF vs SSHP.

**Methods:**

Retrospective case note review of 60 patients who underwent anterior SSF for prolapse between 2009-2017 was performed. Preoperative and postoperative symptoms and findings were recorded. Anterior SSF involved an anterior vaginal incision and paravaginal access to the ligament for dissection and fixation to either the cervix or vault.

**Results:**

SSF was performed in 39 patients, out of which 8 underwent vaginal hysterectomy concomitantly. SSHP for uterine prolapse was performed in 21 patients. There were no cases of recurrent apical prolapse in the cohort at mean follow-up of 1 year. No intra-operative visceral injuries were observed. Recurrence of anterior wall prolapse and postoperative voiding dysfunction was observed in 8.3% and short-term buttock pain in 6.6% of patients.

**Conclusion:**

Anterior approach SSF and SSHP is a safe and effective technique for apical prolapse and is the recommended route when posterior vaginal surgery is not required.

## Introduction

Adequate apical support is essential to improve long term efficacy of surgical repair for advanced vaginal wall prolapse (Brubaker et al., 2010). Wider literature suggests higher failure rate of anterior and posterior repairs when the apex is left unsupported (Hsu et al., 2008). Vaginal sacrospinous fixation and hysteropexy (SSF/SSHP) are effective procedures with low recurrence and complication rates (Lantzsch et al., 2001; Kapoor et al., 2017).

The sacrospinous ligament for fixation of apex can be achieved by two approaches. Traditionally the posterior approach via a posterior vaginal wall incision and dissection through the peri-rectal space is utilised. This technique has been well described in the wider literature and is the most commonly adopted approach. The anterior approach is less well described and researched when compared to the traditional posterior approach. Only three studies reporting surgical outcomes after the anterior approach have been described in the wider literature (Winkler et al.,2000; Cespedes.,2000; Goldberg et al., 2001). Goldberg et al. (2001) compared the two approaches and anterior SSF gave overall better results with increased vaginal length and sexual function. To date there have been no reported series of anterior approach SSHP. This technique is particularly helpful in women who do not have posterior prolapse and do not require posterior vaginal incision and dissection.

The aim of this paper is to review and compare the surgical outcomes of the anterior approach following both SSF and SSHP in our single centre over a 8 year period.

## Methods

A total of 571 sacrospinous fixation procedures were performed between 2009 to 2017. Complete medical records were available for 388 patients only. Out of these 168 fixations were performed by two experienced urogynaecology surgeons with similar surgical techniques. Posterior SSF was performed in 108 and the anterior approach undertaken in 60 patients which were included in this review. All anterior approach fixations were undertaken by two surgeons. A postoperative follow-up visit was arranged for all patients at 3 months after surgery. Examination using Pelvic organ Prolapse Quantification (POP-Q) was undertaken either by urogynaecology nurse specialist, urogynaecology fellow or consultant urogynaecologist. Patients were then either discharged back to their General Practitioner or referred for further management depending on symptoms of recurrent prolapse or incontinence. A retrospective case note review to assess recurrence of prolapse symptoms was undertaken at a mean of 24 months after surgery and recurrence within one year of surgery was noted. Main outcome measure was recurrence of apical prolapse and anterior compartment prolapse. Secondary outcome measures were intra-operative complications and postoperative pain and symptoms.

A standard pro forma was used for data collection. Baseline patient demographics included age, parity, BMI, and smoking status and past medical and surgical history were recorded. Intra- operative variables included details of surgery, blood loss, complications. Postoperative variables included recurrence of prolapse, buttock pain, sexual, urinary and bowel symptoms.

All data was entered and analysed using Microsoft excel 2007. All personal data was anonymised before adding to the database. Student t-test was used to compare continuous variables and Fishers exact test to compare categorical variables. A p-value of <0.05 was considered significant.

As this was a retrospective review of an operative procedure which is standard practice at our trust, ethical approval was not required. The principles outlined in the declaration of Helsinki were followed during this review.

## Surgical technique

All procedures were performed under general anaesthesia with patients in the dorsal lithotomy position. Prophylactic antibiotics were given to all patients intra-operatively according to the hospitals microbiology protocol. Foleys catheterisation was done for all patients. The vaginal skin was then infiltrated with 40-60 ml of 0.25 % bupivicaine and 1:200,000 adrenaline (volume dependent on patient weight). If a vaginal hysterectomy (VH) was part of procedure it was performed first. This was followed by a midline anterior vaginal wall incision extending from below mid-urethra to the level above the vault or cervix. Endopelvic fascial dissection from the vaginal epithelium to the level of pubic rami was undertaken bilaterally. Authors routinely perform sacrospinous fixations unilaterally on the right side. After palpating the ischial spine and the right sacrospinous ligament, access to the sacrospinous ligament was achieved by breaking down fibrous tissue in the paravaginal space by tactile perception. Two PDS-1 sutures on the sacrospinous ligament were inserted using the Capio slim suture capturing device (Boston Scientific). To avoid injury to nearby neurovascular structures, suture placement was ensured to be 2-3cm medial to the ischial spine. Ends of sutures were then passed as a pulley (by tying one end of suture to itself) through the mucosa of the vaginal vault or cervix at the level of uterosacral ligaments or its remnants. Anterior fascial repair was then performed using a delayed absorbable suture (PDS 2-0 or Vicryl-0). Excess vaginal skin was excised if required and skin closed with continuous closure using Vicryl 0. Pulley sutures were then tied down using 5-6 knots on the fixed end and pulling on the free end ensuring no gap was left between knots. Ends were then cut short to about 3cm length. A per-rectal examination was performed at the end of procedure to ensure no rectal injury had occurred.

Postoperatively NSAIDS and paracetamol were given for regular analgesia and opioid analgesics if required. The catheter was routinely removed the following day and a post void bladder scan was performed to check for voiding dysfunction. If patients retained >100-150 ml volume after three voids re-catheterisation for another week was advised. Patients were discharged with laxatives and advised regarding physical activity.

## Results

**Figure 1:**
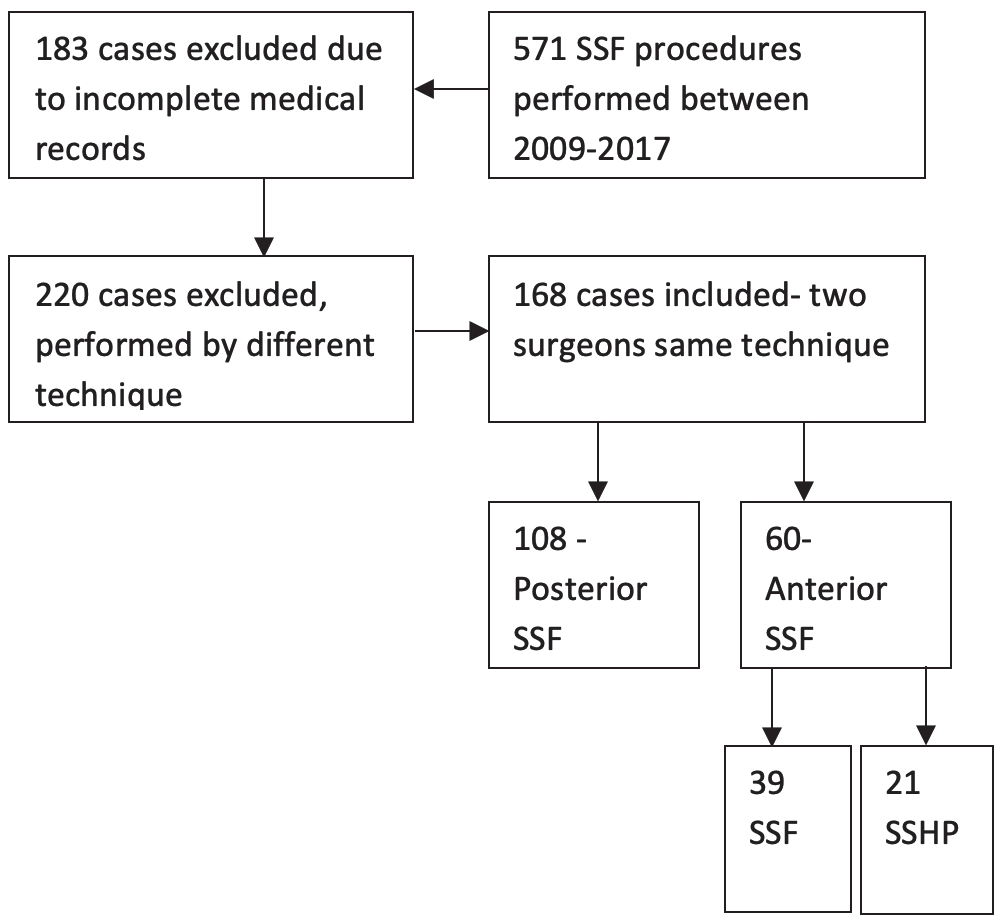
— Number of included cases in each group.

All patients had apical prolapse staged at POP-Q stages 3-4 prior to surgery. SSF of the vault was performed for vault prolapse in 39 patients, out of which 8 underwent vaginal hysterectomy concomitantly, the remaining 31 patients had post hysterectomy vault prolapse. SSHP for uterine prolapse was done in 21 patients. Figure 1 shows the number of included cases in each group. Table I shows a comparison of baseline characters for both groups. There were no statistical differences in the age, BMI, parity, smoking status and previous prolapse surgery between the SSF and SSHP groups.

**Table I:** Baseline variables for SSF and SSHP groups.

	Anterior SSF for vault prolapse (n=39) ^a^	Anterior SSHP for uterine prolapse (n=21)	p-value
Age median, range	67 (48-81)	66.5(35-86)	0.38
Parity median, range	2 (1-4)	2 (1-5)	0.34
BMI median, range	26 (20-37.7)	26 (17-33.6)	0.15
Smoking status, n (%)	1 (2.5)	3 (14.2)	0.11
Previous prolapse surgery n (%)	11(28)	2 (9.5)	0.06

^a^ 8 patients had VH +SSF

There were no organ injuries and no intra- operative complications in either SSF and SSHP groups. All patients in both groups reported being satisfied with results of repair at three months follow-up. POP-Q examination did not reveal any anatomical recurrences of apical or anterior prolapse. There were no reported cases of recurrence of apical prolapse at 1 year. Five patients (2 in SSF group and 3 in SSHP group) had symptomatic anterior wall prolapse (8.3%) within 1 year of surgery and underwent subsequent anterior repairs.

There were no reported cases of any suture erosion in either group. Postoperative voiding dysfunction (VD) was seen in 7.6% and 4.7% of patients in SSF and SSHP groups respectively which was resolved within one week in all, however no long-term voiding problems were seen. On the follow-up visit only four patients (6.6%), all in SSF group, reported that they experienced buttock pain, which resolved with NSAIDS. There were no cases of dyspareunia in our series. Three patients (5%) required subsequent surgery for stress incontinence. Table II shows comparison of postoperative complications between SSHP and SSF groups. There was no statistical difference in outcomes of the two groups. Table III compares outcomes between subgroup of patients undergoing SSF+VH with SSHP. Risk of buttock pain was lower in SSHP group and statistically significant, however there was no difference in other variables.

**Table II:** Comparison of outcomes and complications between SSF and SSHP groups at 1 year.

	Anterior SSF for vault prolapse (n=39) *	Anterior SSHP for cervical/uterine prolapse (n=21)	p-value
Median Blood loss ml	50 (50-200)	50 (50-200)	0.31
Post-op voiding dysfunction	3(7.6%)	2 (4.7%)	0.66
Recurrent apical prolapse	0	0	-
Recurrent anterior prolapse requiring surgery	2 (5.1%)	3 (14.2%)	0.66
Buttock pain	4 (10.2%)	0	0.28
Postop SUI requiring surgery	1 (2.5)	2 (9.5)	0.29

**Table III:** Comparison between subgroups of SSF with vaginal hysterectomy (VH) and SSHP at 1 year.

	Anterior SSF + VH for vault prolapse (n=8)	Anterior SSHP for cervical/ uterine prolapse (n=21)	p-value
Median Blood loss ml	50 (50-200)	50 (50-200)	0.14
Post-op voiding dysfunction	1 (12.5%)	2 (4.7%)	1
Recurrent apical prolapse	0	0	-
Recurrent anterior prolapse requiring surgery	0	3 (14.2%)	0.5
Buttock pain	3 (37.5%)	0	0.01 *
Postop SUI requiring surgery	0	2 (9.5)	1

Table IV shows success and complication rates for all patients in comparison with previous anterior approach publications. No cases of apical prolapse recurrence were seen in our series as compared to previous data. Mean blood loss was less with our technique than previously reported. Rates of anterior prolapse recurrence were comparable to previous reports. Buttock pain was also less pronounced in our results as compared to previous data.

**Table IV:** Comparison of outcomes and complications with previous anterior approach series.

	Our review (SSF+SSHP) N= 60 (%) (12 month Follow-up)	Cespedes (SSF only) N=28 (2000) (17 month Follow-up) N=28 (%)	Goldberg SSF Only N=76 (2001) (39 month Follow-up) N=76 (%)	Winkler SSF only N=75 (2000) (8.5 month Follow-up) N= 75(%)
Bilateral SSF	No	Yes	No	No
Median Blood loss ml (range)	50 (50-200)	160	-	-
Post-op voiding dysfunction	5 (8.3)	1 (4)	-	-
Recurrent apical prolapse	0	1 (4)	-	5 (6.6)
Recurrent anterior prolapse requiring surgery	5 (8.3)	2 (8)	-	2 (2.6)
Buttock pain	4 (6.6)	2 (8)	-	-
Postop SUI requiring surgery	3 (5)	-	-	1 (1.3)

## Discussion

Women undergoing vaginal apical prolapse repair are almost 31- 63% likely to experience recurrence which is almost twice the likelihood of recurrence after a sacrocolpopexy (23%) (Maher et al.,2016). All 3 RCT including SSF technique in the Cochrane systematic review were using the standard posterior approach. To date no RCT’s comparing anterior vs posterior SSF/SSHP or anterior SSF/SSHP with other procedures have been published in the literature. In our cohort of patients undergoing anterior SSF and SSHP there were no cases of short- term apical repair failure. This is in keeping with other short-term results published by earlier RCT for standard posterior approach (Dietz et al.,2010; Detollenaere et al., 2015). A study reporting long term (2-15yrs) follow-up by Aigmueller et al (2008) documented the apical failure rate after posterior approach SSF to be 7% (Aigmueller et al., 2008) Small case series on bilateral anterior approach SSF by Cespedes (2000) reported a recurrence rate of 4% at mean follow-up of 17 months. Our anterior approach technique for both vault and uterine prolapse shows lower risk of recurrence with no reported short-term apical recurrences.

Reported rates of anterior prolapse following posterior approach SSF are variable. Some authors have reported rates as high as 47 % for SSHP (Detollenaere et al., 2015). Longer term reported risk following SSF are as high as 29% at 2-15 years follow-up (Aigmueller et al., 2008).

Others have reported much lower rates of 8.1 % at mean follow-up of 8 years (Lantzsch et al.,2001). Reported rates of cystocele after anterior SSF are 7-9% (Winkler et al.,2000; Cespedes 2000). In our cohort cystocele recurrence was 8.7% which is much lower than the rates documented with the standard posterior approach and comparable to other reported anterior approach series. The higher rates reported for posterior approach SSF as compared to anterior technique support theoretical risk of greater exposure of anterior wall to peritoneal pressures after posterior SSF (Goldberg et al., 2001).

Meta-analysis and RCT have revealed no difference in cystocoele recurrence between vaginal hysterectomy in comparison to posterior SSHP for apical prolapse (OR 1.12) (Kapoor et al.,2017; Detollenaere et al., 2015). We did not find any statistical differences in cystocoele recurrences between our cohort of anterior SSHP and SSF. There was also no difference in outcome between SSHP and VH + SSF groups.

The overall rate of buttock pain in our cohort was 6.6%. All cases were transitory in the immediate postoperative period and resolved within 3 months. Moreover, there were no reported cases of pain in SSHP group. This is much lower when compared to previous studies with posterior approach, have reporting rates of 7.5% for SSF and 9 % for SSHP (Lantzsch et al., 2001; Detollenaere et al., 2015). Cespedes (2000) reported a rate of 8% in their series of bilateral anterior approach SSF in 28 patients.

VD in our cohort was seen in 8.3% of patients. None of them required a catheter beyond one week postoperatively. Variable rates of VD have been reported after posterior SSF ranging from 5-15 % (Lantzsch et al., 2001; Detollenaere et al., 2015). VD after anterior SSF has been reported by Cespedes (2000) to be 4% in their case series. These variable rates are also likely to be affected by different definitions of VD.

Surgery for stress incontinence was required in 5% of our cohort and no differences were found when comparing SSF and SSHP with or without VH. Systematic review of RCT showed similar results with no evidence of increased need of SUI surgery following vaginal procedures (2-16%) as compared to sacrocolpopexy (Maher et al., 2016).

There were no cases of ureteric injury in our review and the authors routinely do not perform cystoscopy post procedure to check for ureteric patency. Reported rates of partial ureteric obstruction with posterior approach are 5.5% (Lantzsch et al., 2001).

Our data suggest that this technique is useful in clinical practice for apical prolapse along with anterior vaginal prolapse with good short-term results.

Although our observation suggests anterior approach SSF and SSHP to be efficacious and safe procedures, there are limitations including the small size of our study, the retrospective data analysis and lack of longer-term follow-up data. Moreover, due to small case numbers, potential confounders and bias including risk for prolapse recurrence and previous surgeries were not taken into account during statistical data analysis. A further long term follow-up study as well as a comparative study with the posterior approach technique including multivariate analysis would allow more definitive conclusions to be withdrawn.

## Conclusion

Anterior sacrospinous fixation and sacrospinous hysteropexy are efficacious and safe procedures for apical compartment prolapse. Our series highlights the low complications rates as well as high success rates and to our knowledge is the first series to report outcomes for anterior approach SSHP. Further comparative studies and RCTs are needed before definitive recommendations can be made, however the preliminary results suggest this is a safe procedure with minimal complications and lower frequency of prolapse recurrence when compared with other techniques.

## References

[1] Aigmueller T, Riss P, Dungl A (2008). Long-term follow-up after vaginal sacrospinous fixation: patient satisfaction, anatomical results and quality of life.. Int Urogynecol J.

[2] Brubaker L, Maher C, Jacquetin B (2010). Surgery for pelvic organ prolapse.. Female Pelvic Med Reconstr Surg.

[3] Cespedes RD (2000). Anterior approach bilateral sacrospinous ligament fixation for vaginal vault prolapse.. Urology.

[4] Detollenaere RJ, den Boon J, Stekelenburg J (2015). Sacrospinous hysteropexy versus vaginal hysterectomy with suspension of the uterosacral ligaments in women with uterine prolapse stage 2 or higher: multicentre randomised non-inferiority trial.. BMJ.

[5] Dietz V, van der Vaart CH, van der Graaf Y (2010). One-year follow-up after sacrospinous hysteropexy and vaginal hysterectomy for uterine descent: a randomized study.. Int Urogynecol J.

[6] Goldberg RP, Tomezsko JE, Winkler HA (2001). Anterior or posterior sacrospinous vaginal vault suspension: long-term anatomic and functional evaluation.. Obstet Gynecol.

[7] Hsu Y, Chen L, Summers A (2008). Anterior vaginal wall length and degree of anterior compartment prolapse seen on dynamic MRI.. Int Urogynecol J Pelvic Floor Dysfunct.

[8] Kapoor S, Sivanesan K, Robertson JA (2017). Sacrospinous hysteropexy: review and meta-analysis of outcomes.. Int Urogynecol J.

[9] Lantzsch T, Goepel C, Wolters M (2001). Sacrospinous ligament fixation for vaginal vault prolapse.. Arch Gynecol Obstet.

[10] Maher C, Feiner B, Baessler K (2016). Surgery for women with apical vaginal prolapse.. Cochrane Database Syst Rev.

[11] Winkler HA, Tomeszko JE, Sand PK (2000). Anterior sacrospinous vaginal vault suspension for prolapse.. Obstet Gynecol.

